# Outpatient Laparoscopic Cholecystectomy

**DOI:** 10.1155/1994/70629

**Published:** 1994

**Authors:** R. Smith, D. Kolyn, R. Pace

**Affiliations:** 1 Department of Surgery Queens University Kingston General Hospital Kingston Ontario Canada; 2 c/o Douglas 3 Kingston General Hospital Kingston Ontario K7L 2V1 Canada

## Abstract

Outpatient Laparoscopic Cholecystectomy was attempted in 98 patients selected from 266 patients presenting for elective cholecystectomy (37%). Two patients required admission following conversion to “open” Cholecystectomy, one patient was admitted for observation because of a technically difficult Laparoscopic Cholecystectomy and 16 patients were admitted because of refractory nausea and vomiting in the early post-operative period. Seventy-nine patients (81%) were able to be discharged home within 4 to 6 hours of surgery, with only one patient requiring readmission to hospital because of the onset of nausea and vomiting. There were no post-operative complications attributable to the outpatient experience. We believe this approach to elective gallbladder pathology can be safely accomplished in selected patients and will be increasingly utilized in the future.


*HPB Surgery*, 1994, Vol. 7, pp. 261–264
Reprints available directly from the publisher
Photocopying permitted by license only

© 1994 Harwood Academic Publishers GmbH
Printed in the United States of America

### OUTPATIENT LAPAROSCOPIC CHOLECYSTECTOMY

R. SMITH, D. KOLYN and R. PACE

*Department of Surgery, Queens University, Kingston General Hospital, Kingston, Ontario, Canada*

Outpatient Laparoscopic Cholecystectomy was attempted in 98 patients selected from 266 patients presenting for elective cholecystectomy (37%). Two patients required admission following conversion to "open" Cholecystectomy, one patient was admitted for observation because of a technically difficult Laparoscopic Cholecystectomy and 16 patients were admitted because of refractory nausea and vomiting in the early post-operative period. Seventy-nine patients (81%) were able to be discharged home within 4 to 6 hours of surgery, with only one patient requiring readmission to hospital because of the onset of nausea and vomiting. There were no post-operative complications attributable to the outpatient experience. We believe this approach to elective gallbladder pathology can be safely accomplished in selected patients and will be increasingly utilized in the future.

KEY WORDS: Laparoscopic Cholecystectomy, Outpatient Surgery

Laparoscopic Cholecystectomy has become accepted worldwide as the procedure of choice for managing elective gallbladder pathology[1]. Our experience with the first 100 patients undergoing this procedure at our institution demonstrated that most patients can undergo the procedure with few complications and a post-operative hospital stay of less than two days on average. These results raised the possibility of true outpatient Laparoscopic Cholecystectomy.

Outpatient Laparoscopic Cholecystectomy, in addition to the obvious economic advantages to the hospital, has substantial benefits to the patient. The patient has the advantage of recuperating in familiar surroundings with a dedicated caregiver who can respond to their individual needs without distractions from other patients as occurs on most surgical wards. We undertook the following study to prospectively evaluate the safety of outpatient Laparoscopic Cholecystectomy.

### PATIENT SELECTION AND METHODS

All patients presenting to the senior authors' clinic between June 1991 and July 1992 were considered for outpatient Laparoscopic Cholecystectomy. Patients were excluded from outpatient consideration if they were elderly, or had significant co-morbid disease. Selected patients had to be highly motivated and desire the procedure to be done as an outpatient. Patients were also required to have a

Presented at The World Association of Hepato-Biliary Surgery Meeting in Hong Kong, June 1992.

Address correspondence to: R.F. Pace M.D. F.R.C.S.(C), c/o Douglas 3, Kingston General Hospital, Kingston, Ontario, Canada K7L 2V1

reliable caregiver in attendance in the early post-operative period. Patients were also required to spend the first evening in the city so that they were close to the hospital should complications arise.

To be discharged from hospital, the patients had to have an uncomplicated operative procedure, with a smooth initial recovery. Any patient with significant post-operative pain or who had severe nausea and vomiting was admitted to the hospital.

Patients were brought to the hospital on the day of surgery and readied in the outpatient surgery unit. Prophylactic antibiotics were given to all patients pre-operatively and a single dose of post-operative antibiotics was given if bile was spilled during the operative procedure. Patients were asked to void just prior going to the operating room. Urinary bladder decompression was not performed routinely prior to abdominal insufflation. Nasogastric tubes were inserted only if a gas-filled stomach obscured visualization of Calot's triangle. Standard dissection techniques using electrocautery were used. Intraoperative cholangiograms were performed selectively. All trochar sites were infiltrated with Bupivicaine Hydrochloride 0.5% and Adrenaline 1:10,000 at the completion of surgery to assist with post operative pain control.

Post-operatively, all patients were monitored in the outpatient surgery unit for 4 to 6 hours. Metaclopramide HCl 10 mg. IV was given to assist with post operative nausea and vomiting. Analgesics and antiemetics were administered on a PRN basis by the nursing personnel. Patients were assessed by the surgical team prior to discharge. A prescription for oral narcotic analgesics was given to manage post-operative pain, and the patients were discharged to be reassessed in clinic two weeks later.

Demographic information on all patients undergoing Laparoscopic Cholecystectomy was gathered in a prospective manner. Operative findings and complications were recorded in a computerized data bank. Comparisons were made between patients undergoing outpatient laparoscopic cholecystectomy and a concurrent cohort of patients undergoing the same procedure as inpatients.

### RESULTS

Between June 1991 and July 1992, 266 patients underwent Laparoscopic Cholecystectomy. 98 (37%) agreed to the outpatient approach at the time of the initial clinic visit. Demographic data is presented in Table 1. Female to male ratio was 4:1 in both groups. Outpatients were in general younger (mean age 40 years versus 52 years). Fewer outpatients had undergone previous abdominal surgery (38% versus 50%). This did not affect the operative technique in that almost all patients had successful pneumoperitoneum obtained with the veress needle (98% vs. 95% inpatients versus outpatients).

Seventeen (17%) outpatients had acutely inflamed or fibrotic gallbladders. In comparison 53 (32%) inpatients had chronically scarred gallbladders. Two outpatients required conversion to "open cholecystectomy" while 14 inpatients required conversion. Operative time was similar in both groups (48 ± 15 min for outpatients, 42 ± 14 min for inpatients).

Of the remaining 96 outpatients having a successful Laparoscopic Cholecystectomy, 16 patients required admission for control of nausea and one

**Table 1** Comparison between 166 patients undergoing planned inpatient Laparoscopic Cholecystectomy and 98 patients undergoing attempted outpatient Laparoscopic Cholecystectomy between June 1991 and July 1992

| | *168 Inpatients* | *98 Outpatients* |
|---|---|---|
| Women: Men | 133:35 (4:1) | 78:20 (4:1) |
| Age Range | 20-87 yrs | 18-69 yrs |
| Mean Age | 52±16 yrs | 40±13 yrs |
| Prv. Abd. Sx. | 83 (50%) | 37 (38%) |
| Veres Insuff. | 159 (95%) | 96 (98%) |
| Fibrosis/Hydrops | 53 (32%) | 17 (17%) |
| Conversions | 14 (8%) | 2 (2%) |
| Operative Time | 48±15 min. | 42±14 min. |
| Post-Op. Stay | 2.9±2.3 days | 0.3±0.8 days |

patient was admitted for observation after an aberrent hepatic artery was divided during the operative procedure. One patient was discharged from the day surgery unit, but required admission shortly thereafter because of the onset of protracted nausea with vomiting. Of the 20 "failed" patients, the mean postoperative stay was 1.5 days with a range of 1 to 5 days.

The remaining 78 outpatients (80%) were discharged without incident. Two patients experienced minor umbilical wound infections, and one patient required an elective ERCP & ES for a retained common bile duct stone. The mean postoperative stay of all 98 patients who desired an outpatient procedure was 0.3 ± 0.8 days while the mean postoperative stay of the 168 inpatients operated on during the same time interval was 2.9 ± 2.3 days. When reviewed in the clinic at two weeks, all patients were delighted with the outpatient experience and expressed no criticism with respect to their care or recovery.

### DISCUSSION

Outpatient Laparoscopic Cholecystectomy is certainly feasable, and we believe can be safely accomplished in selected patients. As we became comfortable with this approach, the percentage of patients having outpatient surgery increased so that 48% of our last 166 patients had an attempted outpatient Laparoscopic Cholecystectomy. Of all patients willing to have their surgery in this fashion, 80% were able to be successfully discharged within 4 to 6 hours of surgery, without complications related to the outpatient approach. Outpatient open Cholecystectomy has been reported, but we believe that it will not be as acceptable to most surgeons or patients as is the Laparoscopic approach[2].

The outpatient experience is not only safe and improves utilization of hospital beds, but it also appears to be preferred by our selected group of highly motivated patients. They feel that they receive good care and enjoy the freedom of their own home and the comfort of their own bed. Their pain control is better, and they can get analgesics on demand instead of waiting for the nurse to dispense it. Post operative nausea remains the major stumbling block to increase the applicability of outpatient laparoscopic cholecystectomy. Further study, with respect to anaesthetic technique and use of narcotic analgesic may be warranted to reduce this problem.

### SUMMARY

Outpatient Laparoscopic Cholecystectomy is applicable in 30% to 50% of patients undergoing elective cholecystectomy. Fully 80% of those patients attempted in this fashion can be successfully discharged within six to eight hours of surgery, and can expect a smooth recuperation at home. We believe that outpatient Laparoscopic Cholecystectomy is a safe and viable alternative to the inpatient approach in selected patients, and that it will become increasingly utilized as fiscal restraints further limit the access to hospital beds.

### *References*

1. Schirmer, B., Edge, S., Dix, J., Hyser, M., Hanks, J. and Jones, R. (1991) Laparoscopic Cholecystectomy — Treatment of Choice for Symptomatic Cholelithiasis. *Ann. Surg.*, **213**(6), 665–667
2. Saltzien, E.C., Mercer, L.C., Peacock, J.B. and Dougherty, S.H. (1992) Outpatient Open Cholecystectomy. *Surg. Gyn. & Obst.*, **174**(3), 173–175

*(Accepted by S. Bengmark 23 April 1993)*

